# Enacting partner specificity in legume–rhizobia symbioses

**DOI:** 10.1007/s42994-024-00193-1

**Published:** 2024-12-23

**Authors:** Xiaocheng Yu, Hongyan Zhu

**Affiliations:** 1 Department of Plant and Soil Sciences, University of Kentucky, Lexington, KY 40546 USA; 2 Division of Biological Sciences, The University of Chicago, Chicago, IL 60637 USA

**Keywords:** Legumes, Nodulation, Nitrogen fixation, Symbiosis specificity

## Abstract

Legumes, such as peas, beans, and alfalfa, have evolved a remarkable ability to establish root nodule symbioses with nitrogen-fixing soil bacteria to fulfill their nitrogen needs. This partnership is characterized by a high degree of specificity, occurring both within and between host and bacterial species. Consequently, nodulation capacity and nitrogen-fixing efficiency vary significantly among different plant–bacteria pairs. The genetic and molecular mechanisms regulating symbiotic specificity are diverse, involving a wide array of host and bacterial genes and signals with various modes of action. Understanding the genetic basis of symbiotic specificity could enable the development of strategies to enhance nodulation capacity and nitrogen fixation efficiency. This knowledge will also help overcome the host range barrier, which is a critical step toward extending root nodule symbiosis to non-leguminous plants. In this review, we provide an update on our current understanding of the genetics and evolution of recognition specificity in root nodule symbioses, providing more comprehensive insights into the molecular signaling in plant–bacterial interactions.

## Introduction

Nitrogen is essential for the survival of all living organisms on earth. It is a vital component of amino acids that make up proteins and nucleic acids that form RNA and DNA. Humans acquire their nitrogen nutrient from dietary proteins, sourced either directly from plants or through the consumption of animals that feed on plants. Plants derive their nitrogen mostly from the soil. However, the depletion of soil nitrogen in agricultural ecosystems poses a significant constraint on plant growth.

Even though dinitrogen gas makes up approximately 78% of the atmosphere, plants cannot directly utilize it due to a strong triple bond holding together the two nitrogen atoms. Consequently, atmospheric nitrogen must be converted to ammonia or nitrate for plant assimilation, a process known as nitrogen fixation. Nitrogen fixation can be achieved through both biological and non-biological means. Modern agricultural practices have heavily relied on synthetic nitrogen fertilizers created through the Haber–Bosch process. While these fertilizers have played a crucial role in global food production, their production entails substantial consumption of finite fossil fuels. Moreover, more than half of the applied nitrogen fertilizers escapes into the environment through leaching, denitrification, and surface volatilization, contributing significantly to environmental pollution and exacerbating climate change (Zhang et al. 2021a).

Biological nitrogen fixation is naturally occurring in terrestrial ecosystems, primarily facilitated by soil microorganisms that utilize the enzyme nitrogenase to convert atmospheric nitrogen into ammonia. These microorganisms encompass heterotrophic free-living soil bacteria, bacteria engaged in associative relationships with plants, and *Rhizobium* and *Frankia* bacteria that form root nodule symbioses with legumes and actinorhizal plants, respectively (Pankievicz et al. 2019). The legume–rhizobia symbiosis represents an efficient and sustainable nitrogen-fixing mechanism, harnessing solar energy through plant photosynthesis. Globally, this symbiotic relationship contributes more than half of the nitrogen produced by the chemical fertilizer industry (Herridge et al. 2008).

The development of legume–rhizobia symbioses represents a multifaceted and tightly regulated process (Oldroyd et al. 2011, 2013). It commences with intricate chemical dialogues between the interacting partners. In nitrogen-deprived soils, legume roots release (iso)flavonoids into the rhizosphere, attracting rhizobia towards the roots while simultaneously triggering the expression of rhizobial nodulation (*nod*) genes responsible for producing lipo-chitooligosaccharides (LCOs), known as nodulation (Nod) factors. Recognition of Nod factors by specific host receptors initiates downstream signaling cascades, culminating in root hair infection, nodule formation, and subsequent rhizobia infection and release within the nodule. One of the earliest host responses involves the curling of root hairs to encapsulate the attached bacteria within an infection pocket, where root cell invaginations initiate the formation and elongation of tubular structures called infection threads. Through these transcellular infection threads, bacteria proliferate and enter the dividing cortical cells of the nodule primordium via an endocytosis-like process. Within nodule cells, bacteria are enveloped in organelle-like structures termed symbiosomes, derived from invaginations of the host cell membrane. Within the confines of the symbiosome, bacteria differentiate into nitrogen-fixing bacteroids (Haag et al. 2013). While root hair infection is the typical, tightly regulated entry point for rhizobia in most legumes, some subtropical legumes, such as peanuts (*Arachis hypogaea*) and the non-legume *Parasponia*, are infected intercellularly through natural cracks or wounds in the root surface, which may involve distinct signaling pathways (Becking 1992; Bhattacharjee et al. 2022; Boogerd and van Rossum 1997; Montiel et al. 2021).

The development of symbiotic nodules involves the coordinated differentiation of both plant nodule cells and bacterial cells. Legume nodules are classified into two primary types: indeterminate and determinate (Hirsch 1992; Nap and Bisseling 1990). Plants, such as peas, clovers, and *Medicago*, form indeterminate nodules, which originate from cell divisions in the inner cortex and have a persistent apical meristem. The nodules are cylindrical and exhibit a developmental gradient from apex to base, which can be divided into distinct zones: a growing meristem zone at the tip, an infection zone, a nitrogen fixation zone, an interzone between the infection and nitrogen fixation zones, and a senescence zone. In contrast, legumes, such as soybeans and common beans, form determinate nodules, which arise from cell divisions in the middle or outer cortex, lack a persistent meristem, and are spherical in shape. The cell divisions in determinate nodules stop at early stages, and the mature nodule develops through cell enlargement, with infected cells synchronously progressing to the nitrogen-fixing stage. In both types of nodules, symbiotic nodule cells undergo genome endoreduplication, leading to polyploidization and cell enlargement. Concurrently, the infected bacteria differentiate into nitrogen-fixing bacteroids, which can be either terminal or reversible depending on the host but independent of the nodule type. Terminal differentiation is characterized by genome endoreduplication, cell elongation, increased membrane permeability, and loss of reproductive ability, while reversible differentiation maintains cell size and DNA content and can return to the free-living state (Haag et al. 2013; Kereszt et al. 2011; Oldroyd et al. 2011). Bacteroids exhibit significant changes in their transcriptome, cell surface structure, and metabolic activities compared to free-living bacteria, making them well-adapted to the intracellular environment and specialized for nitrogen fixation and nutrient exchange (Haag et al. 2013; Mergaert et al. 2006; Prell and Poole 2006).

Legumes and rhizobia exhibit remarkable phylogenetic diversity, such that specific legumes form symbiotic relationships with distinct groups of rhizobial bacteria, and vice versa (Wang et al. 2018a). This specificity is most evident at the species level but also widespread at the genotypic level within the same species. Specificity can manifest at the onset of interactions, permitting a bacterial strain to infect and nodulate certain host plants while excluding others (nodulation specificity). Additionally, incompatibilities may emerge during later stages of nodule development, resulting in significant disparities in nitrogen-fixing efficiency across different plant–bacteria pairings (nitrogen fixation specificity). A comprehensive understanding of the genetic and molecular mechanisms governing symbiotic specificity is essential for devising strategies to genetically manipulate either the host or bacteria to overcome host range barrier, thereby enhancing nodulation capacity and nitrogen fixation efficiency. This review endeavors to update our current comprehension of the evolutionary dynamics of specificity in legume root nodule symbioses.

## Bacterial recognition of host-secreted flavonoids defines compatibility at the initial communication stage

In response to nitrogen deficiency, legume roots exudate flavonoid compounds into the rhizosphere, acting as chemical signals to initiate communication with compatible rhizobia. These flavonoids activate the rhizobial NodD transcription factors, promoting the expression of *nod* genes essential for Nod factor biosynthesis (Long 1996; Peck et al. 2006). NodD proteins in diverse rhizobial species have evolved to respond to distinct flavonoids released by different legumes, and this interaction specificity plays a crucial role in determining partner compatibility (Liu and Murray 2016). For instance, 4,4’-dihydroxy-2'-methoxychalcone (DHMC) from *Medicago* activates NodD1 in *Sinorhizobium meliloti* but not in *Bradyrhizobium japonicum,* the symbiont of soybeans (Banfalvi et al. 1988; Maxwell et al. 1989). Conversely, genistein and daidzein from soybeans induce *nod* gene expression in *B. japonicum* but not in *S. meliloti* (Wu et al. 2024). Consistent with these findings, the transfer of *nodD* genes between rhizobial species can alter the response of the recipient strains to specific flavonoid inducers, thereby influencing their ability to infect host plants. For example, the NodD1 from the broad-host-range strain *Rhizobium* NGR234 can be activated by a wider array of flavonoid inducers; introducing NodD1 from NGR234 into the narrow-host-range strain *Rhizobium leguminosarum* biovar *trifolii* ANU843 enabled the latter to nodulate the non-legume *Parasponia* (Bender et al. 1988).

Root exudates comprise a complex array of flavonoids, making it challenging to pinpoint the specific compounds and their precise timing and locations of synthesis that exert the greatest influence (Liu and Murray 2016). Nonetheless, genetic studies in plants have validated findings from bacterial genetics. In soybeans, genetic silencing of *isoflavone synthase* (*IFS*) reduces the levels of isoflavones (daidzein and genistein) and abolishes nodulation, illustrating the pivotal role of endogenous isoflavones in fostering symbiosis between soybean and *B. japonicum* (Subramanian et al. 2006). Similarly, in *Lotus japonicus*, a chalcone isomerase, CHI4, is crucial for activating NodD1 in *Mesorhizobium loti* R7A, highlighting its specific involvement in triggering *nod* gene expression within root hair infection threads (Kelly et al. 2018). In *Medicago*, both dihydroxyflavone (DHF) and DHMC are *nod* gene-inducing flavonoids, with DHMC being the most potent (Maxwell et al. 1989). Knockdown of two flavone synthases responsible for DHF synthesis resulted in reduced nodulation in *M. truncatula* (Zhang et al. 2007, 2009). DHMC in *Medicago* is produced by the legume-specific chalcone-O-methyltransferases (ChOMTs) (Maxwell et al. 1992, 1993). The *M. truncatula* genome contains multiple *ChOMT* homologs, some of which are specifically expressed in infected root hairs during rhizobial infection and in the infection zone of mature nodules. It was recently demonstrated that ChOMT1 and ChOMT3 are required for DHMC production and indispensable for nodulation (Breakspear et al. 2014; Chen et al. 2015; Wu et al. 2024). In contrast, soybean ChOMT homologs are not induced by *B. japonicum* (Libault et al. 2010), suggesting that DHMC is unlikely to play a role in soybean nodulation.

Despite the well-documented recognition specificity of flavonoid–NodD interactions, experimental evidence for their direct binding remains lacking. Consequently, the precise structural determinants that enable NodD proteins to recognize and respond to specific flavonoid signals are still poorly understood. Investigating the molecular details of flavonoid–NodD binding could identify key structural motifs in flavonoids critical for recognition, which could, in turn, guide the development of flavonoid analogs to better understand specificity. Additionally, profiling flavonoid metabolites across legume species may reveal the diversity of flavonoids involved in nodulation. This knowledge could facilitate the genetic manipulation of both rhizobia and legumes to alter NodD-binding capabilities and flavonoid production, potentially widening the host range of rhizobia. Furthermore, exploring the evolutionary and ecological context of flavonoid–NodD interactions could further enhance strategies for optimizing rhizobial inoculants and improving legume crop productivity.

## Nod factors dictate the host range of rhizobia, functioning as both specificity determinants and master-regulatory inputs

**Fig. 1:**
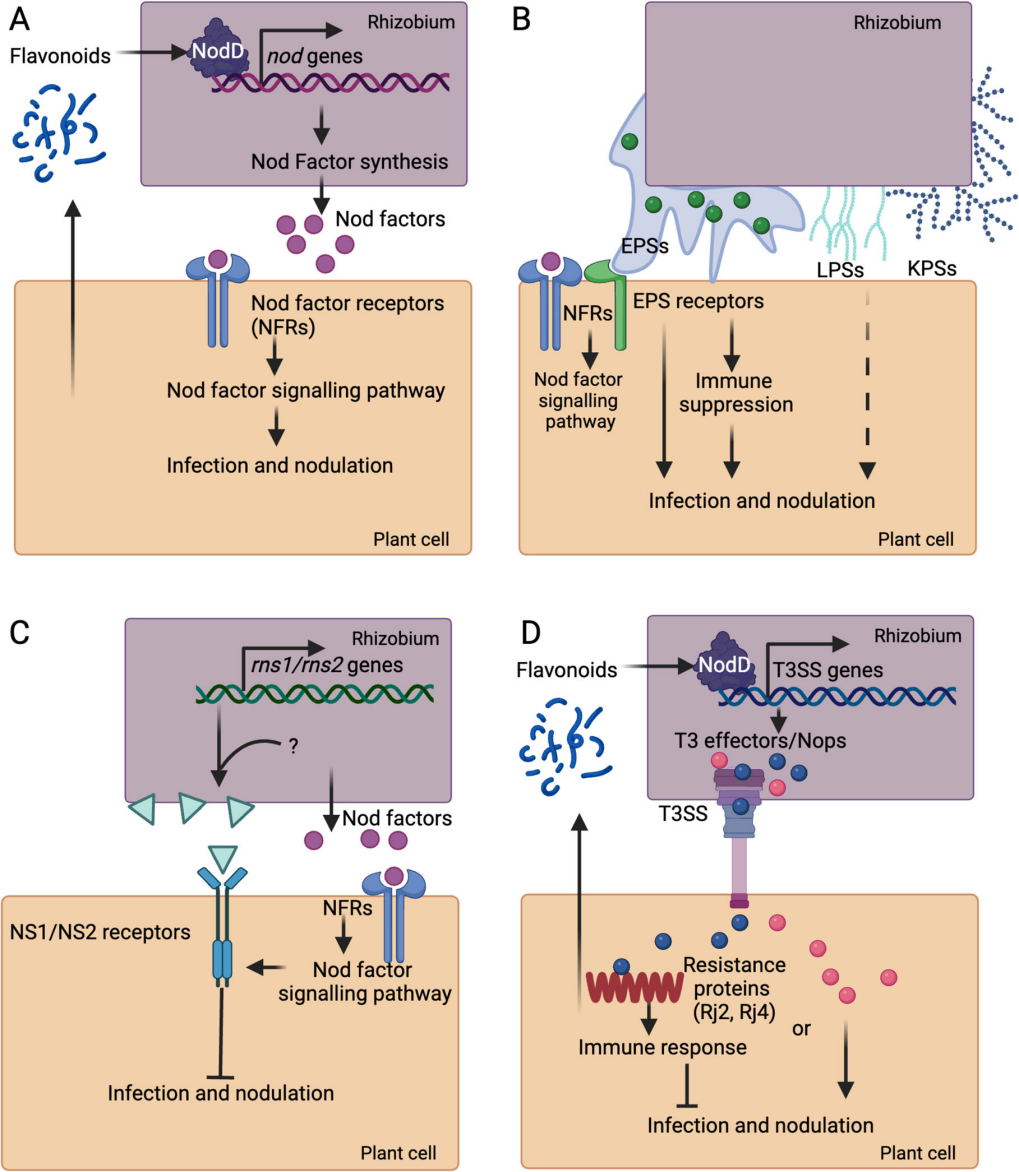
Symbiosis signaling and plant immunity that affect nodulation specificity in the legume–rhizobial symbiosis. **A** Nod Factor biosynthesis and signaling. The interaction between host-secreted flavonoids and bacterial NodD proteins leads to the expression of *nod* genes, resulting in the synthesis of Nod factors (NFs). These NFs are recognized by host Nod factor receptors (NFRs), initiating the nodulation signaling pathway. **B** Role of extracellular polysaccharides. Rhizobia produce extracellular polysaccharides (EPSs), lipopolysaccharides (LPSs), and capsular polysaccharides (KPSs) to facilitate infection during compatible interactions, or to induce immune responses in incompatible interactions. **C** Surface receptor-mediated nodulation specificity in *Medicago. NS1* and *NS2*, both encoding malectin-like domain leucine-rich repeat receptor kinases (MLD-LRR-RLKs), restrict infection and nodulation with *Sinorhizobium* species that possess *rns1* and *rns2* genes, respectively. **D** Effector protein delivery and immune response. Some bacteria employ the Type III secretion system (T3SS) to deliver effector proteins (Nops) that facilitate infection and nodulation. However, in some cases, these molecules are recognized by plant resistance (R) proteins, triggering host immune responses that restrict infection and nodulation. Figure was created with BioRender.com

Recognition of rhizobium-secreted Nod factors by a compatible host plays a pivotal role in initiating nodule organogenesis and bacterial infection (Zipfel and Oldroyd 2017) (Fig. 1A). Nod factors are acylated chitin oligomers with unique chemical modifications determined by species- or strain-specific *nod* genes (Dénarié et al. 1996). This structural diversity is key to the specificity of the legume–rhizobial interaction. Legumes have evolved to recognize specific Nod factors produced by compatible rhizobial species, and this specificity is mediated by the interaction of Nod factors with plant lysine motif (LysM)-containing receptor-like kinases.

In the model legumes *Lotus japonicus* (*Lotus*) and *Medicago truncatula* (*Medicago*), Nod factors are perceived by a pair of LysM receptor kinases that form heterodimers capable of binding Nod factors with high affinity (Arrighi et al. 2006; Broghammer et al. 2012; Limpens et al. 2003; Madsen et al. 2003; Radutoiu et al. 2003). In *Lotus*, the paired receptors are Nod factor receptor 1 (LjNFR1) and LjNFR5, while in *Medicago*, their orthologous counterparts are LysM domain-containing receptor-like kinase 3 (MtLYK3) and Nod factor perception (MtNFP), respectively. The *Lotus* receptor pair recognizes fucosylated Nod factors from *Mesorhizobium loti*, whereas the *Medicago* receptor pair specifically perceives sulfated Nod factors from *Sinorhizobium meliloti*. Expressing *LjNFR1* and *LjNFR5* in *Medicago* enabled nodulation by the *L. japonicus* symbiont *M. loti* (Radutoiu et al. 2007).

Genetic studies also unveiled the specificity of Nod factor recognition within a legume species. For instance, mutations in bacterial *nod* genes in *Rhizobium leguminosarum* lead to alterations in Nod factor composition or structure, resulting in genotype-specific nodulation in pea (*Pisum sativum*) (Bloemberg et al. 1995; Firmin et al. 1993). This host range alteration mirrors allelic variations at the *Sym2/Sym37* locus, an orthologous region of *LjNFR1*, harboring a cluster of LysM receptor kinases. In this case, allelic variation, combined with gene duplication and diversification, contributes to modifications in symbiotic compatibility (Li et al. 2011; Zhukov et al. 2008). Similarly, the presence of a newly evolved chimeric LysM receptor at the *MtLYK3* locus in the *Medicago* genotype R108 enables it to form nodules with the *Sinorhizobium meliloti nodF/nodL* mutant, which produces non-O-acetylated Nod factors; this receptor also plays a crucial role in nodulation by various natural *Sinorhizobium* strains (Luu et al. 2022).

In addition to their role in Nod factor perception, plant LysM-type receptor kinases also recognize short-chain chitin oligomers (CO4-5) and acylated COs produced by mycorrhizal fungi (Myc factors) for symbiosis development (Carotenuto et al. 2017; Maillet et al. 2011). Furthermore, the LysM-type chitin elicitor receptor kinase (CERK) has the capability to detect long-chain chitin oligomers (CO6-8), which are conserved molecular patterns of fungal cell walls, thereby initiating innate immune responses against fungal pathogens. Interestingly, CERK1 orthologs are essential for both arbuscular mycorrhizal (AM) symbiosis and innate immunity in rice and *Medicago* (Feng et al. 2019; Zhang et al. 2015), and the switch of the symbiosis and immune signaling is mediated by competitive binding of CERK1 with respective symbiosis/immune co-receptors or specific interacting proteins (He et al. 2019; Wang et al. 2024; Zhang et al. 2021b, 2024).

LjNFR1 and MtLYK3 are close homologs of CERK-type pattern recognition receptors, originating from a series of duplications in a legume ancestor (De Mita et al. 2014; Rutten et al. 2020). Subsequently, these duplicated genes underwent function diversification, resulting in losing the ability to recognize long-chain chitin oligomers while gaining specificity for Nod factors (Bozsoki et al. 2017; Feng et al. 2019; Gibelin-Viala et al. 2019). This evolution prompts inquiries into the structural modifications required for the receptors to become specific to Nod factors (Bozsoki et al. 2020). The crystal structures of the ligand-binding sites in LjNFR1, MtLYK3, and the CERK receptors exhibit remarkable similarity. The primary structural distinction lies within a small segment in the first of three LysM domains (LysM1) in the Nod factor receptors, which dictates ligand-binding specificity. Across Nod factor receptors in diverse legume species, this segment displays variability, implying evolutionary adaptations for recognizing distinct Nod factor configurations. Conversely, within CERK receptors, this segment remains highly conserved. These molecular signatures enabled the design of ligand recognition motifs tailored for fucosylated or sulfated Nod factors, facilitating the engineering of rhizobium specificity. These findings pave the way for engineering novel functionalities and specificities and potentially facilitate the transfer of the nodulation property to non-legumes (Bozsoki et al. 2020).

## Nodulation specificity mediated by cell surface receptors: beyond Nod factor perception

In addition to Nod factors, rhizobia utilize extracellular components, such as surface polysaccharides and secreted/surface proteins, to modulate their interactions with legume roots (Fig. 1B and C). These extracellular components are crucial for bacterial attachment to plant roots, suppression of host immunity, and protection of rhizobia from plant-secreted antimicrobial peptides (Arnold et al. 2018; Downie 2010; Gibson et al. 2008). Given the diverse variety of surface components produced by different rhizobia, the ability of legume roots to perceive and interact with these signals constitutes another critical checkpoint in symbiosis specificity.

### Surface receptor-mediated perception of exopolysaccharides

Surface polysaccharides, including exopolysaccharides (EPSs), lipopolysaccharides (LPSs), capsular polysaccharides (KPSs) and cyclic glucans, play complex and variable roles in symbiosis (Downie 2010) (Fig. 1B). Among these, EPSs are the most well-characterized. For example, *S. meliloti*, the symbiotic partner of *Medicago*, produces two types of EPSs, succinoglycan (ESP I) and galactoglucan (EPS II), of which EPS I is universally required for bacterial infection (Cheng and Walker 1998). Increased production of succinoglycan enhances nodulation capacity (Jones 2012). However, the role of EPSs in the *Mesorhizobium–Lotus* interaction is more complex: a subset of EPS mutants of *M. loti* R7A displayed severe nodulation deficiencies on *L. japonicus*, while other mutants formed effective nodules (Kelly et al. 2013). Specifically, R7A mutants deficient in producing an acidic octasaccharide EPS could normally nodulate *L. japonicus*, whereas the *exoU* mutants producing a truncated pentasaccharide EPS failed to invade the host. It has been proposed that full-length EPS serves as a signal to compatible hosts, modulating plant defense responses to facilitate bacterial infection, and R7A mutants that produce no EPS might avoid the plant surveillance system, thereby retaining the ability to form nodules. In contrast, strains producing modified or truncated EPSs, however, trigger plant defense responses, resulting in a block of infection (Wightman et al. 2024).

An EPS receptor (LjEPR3) has been identified in *L. japonicus* (Kawaharada et al. 2015, 2017). This receptor is a cell surface-localized protein containing three extracellular LysM domains and an intracellular kinase domain. LjEPR3 binds rhizobial EPS in a structurally specific manner. Interestingly, *LjEPR3* gene expression is contingent on Nod factor signaling, suggesting that bacterial entry into the host is controlled by two successive steps of receptor-mediated recognition of Nod factor and EPS signals. This receptor–ligand interaction supports the notion that EPS recognition plays a role in regulating symbiosis specificity.

*LjEPR3* orthologous genes are widely conserved in the nitrogen-fixing clade of plants that engage in nodulation symbioses with diazotrophic *Frankia* or rhizobia, including both legumes and non-legumes (whether nodulating or non-nodulating) (Dupin et al. 2022). In *M. truncatula*, the *LjEPR3* ortholog, *MtLYK10*, is crucial for the progression of the infection thread to the nodule primordia; however, binding of succinoglycan by MtLYK10 was not observed (Maillet et al. 2020). Intriguingly, the *LjEPR3* ortholog in *Parasponia* species (*EPR*) is a pseudogene due to the insertion of a conserved retrotransposon in the promoter region (Dupin et al. 2022). *Parasponia* is the only lineage of non-legume plants that nodulate with rhizobium. Nevertheless, when the *EPR* gene from two *Trema* species (the sister genus of *Parasponia* which is assumed to have lost the nitrogen-fixing nodulation trait) was transferred to *Parasponia*, it revealed nodulation-specific expression. This suggests that the *EPR* gene functioned in nodulation in the common ancestor of *Parasponia* and *Trema*. It is speculated that loss of this ancestral function may have been instrumental in the microsymbiont switch from *Frankia* to rhizobia in the *Parasponia* lineage, leading to the evolution of a novel crack entry infection mechanism (Dupin et al. 2022).

### Surface receptor-mediated perception of yet unknown ligands

Unlike the intracellular immune receptors (*R* genes)-regulated nodulation specificity in soybeans, which relies on the bacterial type III secretion system and its secreted effectors (described in the next section), the microsymbionts of *Medicago*, primarily *Sinorhizobium meliloti* and *Sinorhizobium medicae*, generally lack such a secretion system. Although *S. meliloti* and *S. medicae* are closely related and share a similar host range, certain *Medicago* species exhibit selective interactions with these two bacterial species (Garau et al. 2005; Rome et al. 1996). Two distinct loci, *NS1* and *NS2*, have been identified in *Medicago* that regulate nodulation specificity with the two *Sinorhizobium* species (Liu et al. 2014, 2022a, b; Yu et al. 2024) (Fig. 1C).

The *NS1* locus impedes infection and root nodule formation by many *S. meliloti* strains (Liu et al. 2014, 2022a, b). It comprises two tandem genes, *NS1a* and *NS1b*, encoding malectin-like leucine-rich repeat receptor kinases (MLD-LRR-RLKs) that control this strain-specific nodulation blockade. Expression of *NS1a* and *NS1b* is induced upon inoculation by both compatible and incompatible *Sinorhizobium* strains, and this gene induction is dependent on Nod factor signaling. Presence, absence, and sequence variations of these paired receptors contribute to the evolution and functional diversification of the *NS1* locus. The bacterial gene *rns1* is required for the activation of NS1-mediated nodulation restriction. *rns1* encodes a type I-secreted protein with parallel beta-helix repeats, commonly found in polysaccharide lyases (Finnie et al. 1998). Approximately half of the nearly 250 sequenced *S. meliloti* strains contain *rns1*, while it is absent in over 60 sequenced strains of *S. medicae*. *S. meliloti* strains lacking functional *rns1* can evade NS1-mediated nodulation blockade (Liu et al. 2022a).

Conversely, the *NS2* locus prevents infection and nodulation by a broad range of *S. medicae* strains (Yu et al. 2024). *NS2* also encodes an MLD-LRR-RLK and is part of a large gene cluster, with homologous clusters in the syntenic genomic regions of both legumes and non-legumes (Yang et al. 2021; Yu et al. 2024). Most sequenced *Medicago* accessions lack allelic copies of *NS2*, suggesting selective pressures to exclude this gene from natural populations. The *Medicago* accession DZA220-H, which carries the *NS2* gene, is classified as *M. truncatula* but likely belongs to *M. littoralis* or is an introgressant derived from it, based on the presence of a 45-kb inversion (rpl20-ycf1) in its plastid genome (Choi et al. 2022). This suggests that the *NS2* gene originated from *M. littoralis*, a species more strongly associated with *S. meliloti* than *S. medicae* (Garau et al. 2005). NS2-mediated resistance to infection depends on *S. medicae*-specific variants of the *rns2* gene, which encodes a putative glycine-rich cell wall structural transmembrane protein (Yu et al. 2024).

Direct physical interactions between NS1a/NS1b and Rns1, and between NS2 and Rns2 have not been detected, suggesting that Rns1 and Rns2 likely are not the direct ligands perceived by the receptor kinases. Instead, they are probably involved in producing metabolic products that are recognized by the receptor kinases. Given that both *NS1* and *NS2* genes encode homologous receptor kinases and exhibit similar expression patterns, one can speculate that their recognition mechanisms might be similar. However, the molecular and biochemical functions of Rns1 and Rns2 are yet to be elucidated. It has been reported that *rns2* of *S. meliloti* (SMc04236) may be a direct target of the ExoS/ChvI two-component signaling pathway, which is crucial for establishing root nodule symbiosis and regulates many free-living bacterial phenotypes, such as exopolysaccharide production, motility, and cell envelope integrity (Chen et al. 2009).

Further research is necessary to elucidate the genetic pathways in the plant and the molecular signals in the bacteria to better understand how receptor-like kinases perceive and transmit bacterial signals, how the signaling pathway interacts with nodulation and/or immunity signaling, and ultimately how this leads to the specialization of host-bacterial symbiosis.

## Rhizobial type III effectors: double-edged swords in symbiosis development

Plants encounter a broad diversity of pathogenic and symbiotic microorganisms in their surroundings, requiring the ability to discern between them and initiate suitable responses. While both pathogens and symbionts share common microbe-associated molecular patterns (MAMPs) capable of triggering host immunity, plants and their microsymbionts have developed mechanisms to suppress or evade these defense responses, thereby promoting symbiosis development (Zipfel and Oldroyd 2017). For instance, bacterial flagellin, a classical MAMP, harbors a conserved 22-amino-acid segment known as flg22, which triggers immune responses in most plants through interaction with the FLS2 receptor (Boller and Felix 2009). The presence of flg22 in rhizobia could render them susceptible to immune activation, potentially impeding symbiosis establishment (Lopez-Gomez et al. 2012). However, the flg22 variants found in rhizobia fail to induce an immune reaction due to the absence of conserved recognition residues (Boller and Felix 2009), suggesting that rhizobial bacteria have evolved mechanisms to evade detection of this MAMP. As discussed in the preceding section, LysM-type receptors such as CERK1 can engage with both immunogenic and symbiotic signals; however, the downstream pathways governing symbiosis and immunity signaling can be separated by their interactions with distinct symbiotic/immune co-receptors or proteins (Bozsoki et al. 2017; Wang et al. 2024; Zhang et al. 2021b). Furthermore, rhizobium-secreted Nod factors have been demonstrated to be able to suppress immunity signaling during symbiosis (Gourion et al. 2015), probably facilitated by the binding of Nod factor receptors with their symbiotic co-receptors, thereby diminishing their interaction with immune co-receptors on the cell surface (Liang et al. 2013; Zhang et al. 2021b).

Similar to pathogenic bacteria, rhizobial bacteria also secrete effector molecules to suppress MAMP-triggered immunity (Deakin and Broughton 2009; Nelson and Sadowsky 2015). These effectors are delivered into the plant cell via bacterial secretion systems, notably the type III secretion system (T3SS), the type IV secretion system (T4SS), and the type VI secretion system (T6SS), with the T3SS being the most extensively studied (Nelson and Sadowsky 2015; Teulet et al. 2022) (Fig. 1D). The prevalence of T3SS varies significantly among rhizobial genera; for instance, most *Bradyrhizobium* strains carry T3SS-encoding genes (Teulet et al. 2020), but only a small portion of the sequenced *Sinorhizobium* strains possess such genes (Sugawara et al. 2013). In many instances, *nod* and T3SS-encoding genes are embedded within symbiotic islands; moreover, the expression of T3SS operons is also reliant on flavonoids and NodD, suggesting a shared evolutionary path aimed at facilitating nodulation and circumventing host immunity (Teulet et al. 2020). Effector proteins secreted by rhizobia T3SSs (T3Es), commonly known as nodulation outer proteins (Nops), often exhibit structural similarities to effectors produced by pathogenic bacteria.

T3Es (Nops) of rhizobia can have positive, negative, or neutral effects on nodulation, depending on the host plant’s genetic background (Kambara et al. 2009; Songwattana et al. 2021). In the absence of recognition, such effectors could promote rhizobial colonization. However, recognition of these effectors by the host immune receptors can trigger immunity, thus restricting rhizobial infection and nodulation (Staehelin and Krishnan 2015). Several dominant genes have been cloned in soybeans that constraint nodulation by specific rhizobial strains (Tang et al. 2016; Yang et al. 2010; Zhang et al. 2021c). These genes encode intracellular immune receptors or defense-related proteins that recognize specific rhizobial T3Es, which exhibit a striking similarity to the 'gene-for-gene' resistance observed in plant-pathogen interactions. Notably, *Rj2* and *Rfg1* are allelic genes that encode a typical Toll-interleukin receptor (TIR) nucleotide-binding site (NBS)-LRR protein (TIR-NBS-LRR), imparting resistance against a spectrum of strains from *Bradyrhizobium japonicum* and *Sinorhizobium fredii*, respectively (Yang et al. 2010). Rj2 recognizes rhizobial NopP from incompatible strains, triggering immune responses that restrict infection and nodulation (Sugawara et al. 2018). Similarly, the *NNS1* gene, also encoding a TIR-NBS-LRR protein, discerns a distinct variant of NopP from *B. japonicum* USDA110, resulting in symbiotic incompatibility (Zhang et al. 2021c). However, the soybean *Rj4* gene, which confers resistance to *Bradyrhizobium elkanii* strain USDA61 via effector-triggered immunity, encodes a thaumatin-like protein instead of an NBS-LRR (Faruque et al. 2015; Tang et al. 2016). The recognition of T3Es by a thaumatin-like protein has not been previously documented. It is plausible that Rj4-mediated nodulation restriction also requires an as-yet-unidentified NBS-LRR-encoding gene. These exemplified instances underscore a pivotal role of effector-triggered immunity in delineating host specificity.

In certain scenarios, nodulation can occur in the absence of Nod factor production or perception (Giraud et al. 2007; Okazaki et al. 2013, 2016). This Nod factor-independent nodulation often depends on the presence of a functional T3SS (Okazaki et al. 2013, 2016). For instance, the nodulation of the soybean genotype Enrei or its *nfr1* mutant by a *nodC* mutant of *B. elkanii* USDA61 requires the type III effector Bel2-5, which is also known to inhibit nodulation in the presence of the *Rj4* allele (Faruque et al. 2015; Ratu et al. 2021). Similarly, various T3Es, such as ErnA and Sup3, have been identified in different *Bradyrhizobium* strains capable of inducing Nod factor-independent nodulation on *Aeschynomene indica* (Camuel et al. 2023; Teulet et al. 2019). ErnA is prevalent among *Bradyrhizobium* strains, whereas Sup3 is found in strains lacking ErnA. Ectopic expression of *ernA* and *sup3* in transgenic roots of *A. indica* leads to spontaneous development of nodule-like structures without inoculation. Both ErnA and Bel2-5 target the host cell nucleus, suggesting roles in transcriptional activation of the nodulation program (Okazaki et al. 2013; Ratu et al. 2021). Although *bel2-5* and *ernA* are necessary for Nod factor-independent nodulation, all strains containing *ernA* and/or *bel2-5* also possess *nod* genes involved in Nod factor synthesis (Ratu et al. 2021; Teulet et al. 2019, 2020). It is also noteworthy that nodules induced by type III effectors, without Nod factor perception, exhibit limited nitrogen fixation capabilities (Camuel et al. 2023). This raises the questions of whether a symbiosis solely dependent on a T3SS truly exists in nature and what roles these T3Es play in canonical Nod factor-dependent symbioses. It is possible that they collaborate with Nod factors to enhance a strain's nodulation capabilities. Alternatively, rhizobia may have evolved T3Es to interact with legumes that are unresponsive to their secreted Nod factors (Teulet et al. 2022).

## Nitrogen fixation specificity: incompatibilities emerging during the later stages of symbiosis development

Beyond the initial recognition stages, specificity can also occur during the later stages of nodule development, resulting in significant variations in nitrogen fixation efficiency across different plant–bacteria pairings. It is not uncommon for some legume–rhizobium pairs to form infected root nodules that are unable to fix nitrogen. The genetic mechanisms governing this specificity are complex and involve both plant and bacterial genes. Additionally, this regulation is dependent on whether the differentiation of nitrogen-fixing bacteroids is terminal or reversible.

**Fig. 2:**
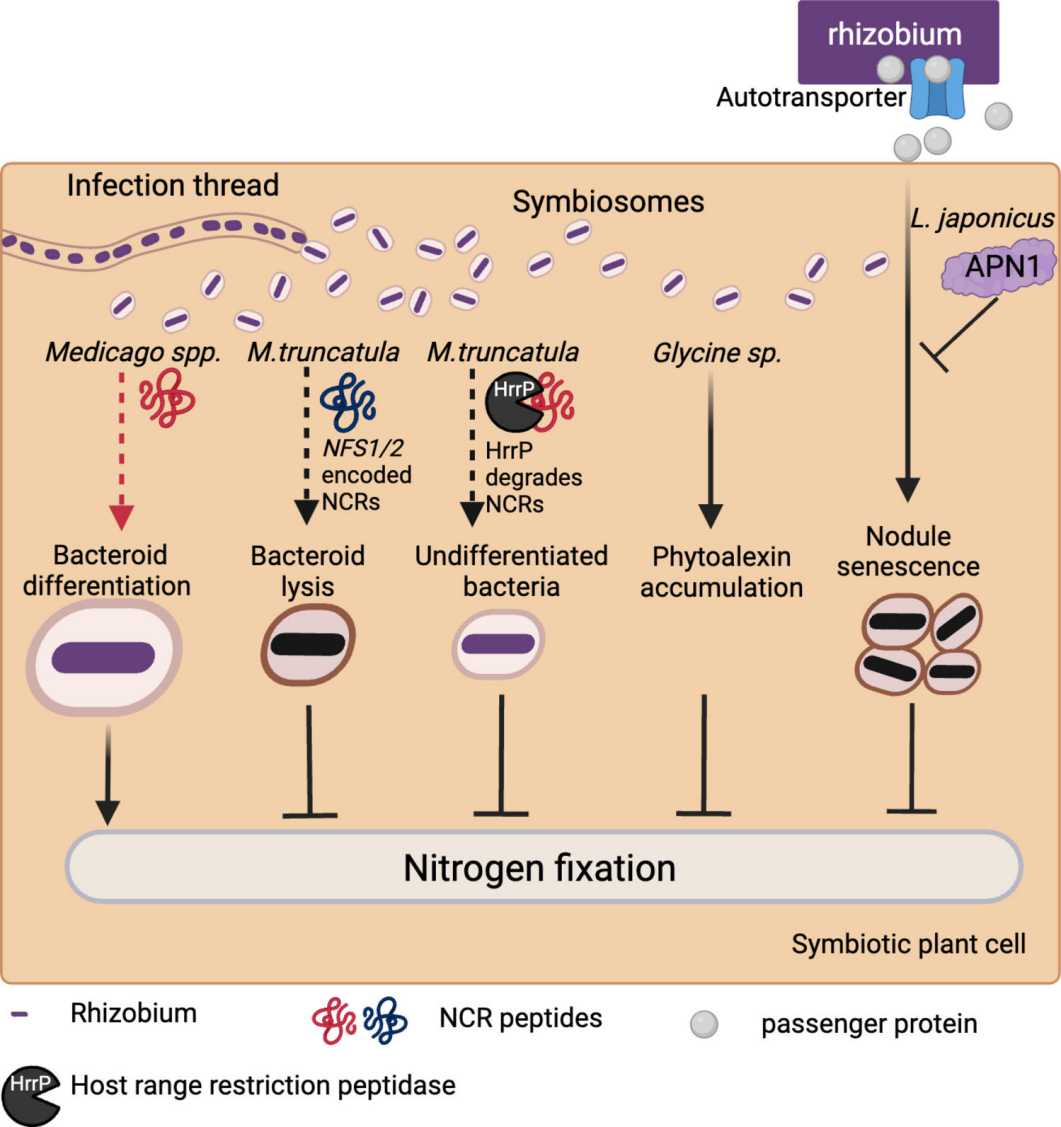
Factors influencing nitrogen fixation specificity in the legume–rhizobial symbiosis. In *Medicago*, NCRs are crucial for bacteroid differentiation and survival within nodule cells, which is essential for effective nitrogen fixation. However, certain rhizobial strains are vulnerable to the antimicrobial activity of specific NCR isoforms, resulting in bacteroid lysis and a failure in nitrogen fixation. The bacterial host range restriction peptidase (HrrP) can degrade NCRs, disrupting bacterial differentiation and impairing nitrogen fixation. In soybeans, incompatible strains can trigger phytoalexin accumulation and hypersensitive responses in nodule cells, which interfere with nitrogen fixation. In *Lotus japonicus*, the aspartic peptidase APN1 can cleave the bacterial passenger protein. This cleavage prevents the induction of nodule senescence, which would otherwise inhibit nitrogen fixation. Figure was created with BioRender.com

Galegoid legumes (belonging to the Inverted Repeat-Lacking Clade or IRLC), such as pea (*Pisum*) and *Medicago* spp., induce their microsymbionts to undergo terminal differentiation to enhance nitrogen fixation efficiency (Oono and Denison 2010; Oono et al. 2010) (Fig. 2). In *Medicago*, and likely in other galegoid legumes as well, terminal bacteroid differentiation is driven by a large family of nodule-specific, cysteine-rich (NCR) peptides (Van de Velde et al. 2010; Wang et al. 2010). These peptides possess antimicrobial properties and are expressed exclusively in infected nodule cells (Montiel et al. 2017). The NCRs are delivered into the symbiosome via a symbiosis-specific secretion complex that recognizes the highly conserved N-terminal signal peptides on NCRs (Wang et al. 2010; Stonoha-Arther and Wang 2018). Numerous NCRs have been shown to be positive regulators of symbiotic development, essential for bacterial differentiation and maintenance of bacterial survival within nodule cells (Horváth et al. 2015, 2023; Kim et al. 2015).

The NCR peptides can also negatively impact symbiotic development in a strain-specific manner (Fig. 2). By leveraging natural variation in *Medicago truncatula*, two genes in the Jemalong A17 genotype, *NFS1* and *NFS2*, were identified that contribute to the nitrogen fixation failure (Fix-) with *S. meliloti* strains Rm41 and A145 (Wang et al. 2017, 2018b; Yang et al. 2017). Both *NFS1* and *NFS2* encode NCR peptides that cause bacteroid lysis and early nodule senescence in an allele-specific and rhizobial strain-specific manner, with their function being influenced by both host and bacterial genetic backgrounds. These findings suggest that NCRs act as a double-edged sword in symbiosis development. On the one hand, the host employs this antimicrobial strategy to promote terminal bacteroid differentiation, prioritizing nitrogen fixation efficiency. On the other hand, some rhizobial strains cannot withstand the antibacterial activity of certain peptide isoforms. Maintaining this balance likely drives the coevolution of both symbiotic partners, leading to the rapid amplification and diversification of NCR genes in galegoid legumes and the development of NCR tolerance mechanisms in their microsymbionts. Consequently, different hosts vary in their deployment of NCR peptide arsenals, and rhizobial strains have varying levels of sensitivity to these peptides.

The tolerance of NCR peptides by *Sinorhizobium* requires the BacA protein (Haag et al. 2011). BacA is a membrane transport protein essential for bacteroid survival within the symbiosome in galegoid legumes (Barriere et al. 2017; Glazebrook et al. 1993; Guefrachi et al. 2015; Karunakaran et al. 2010). Depleting the *bacA* gene in *S. meliloti* Rm2011 is sufficient to convert a Fix + phenotype to Fix − in *Medicago* (DiCenzo et al. 2017). Although the *bacA* orthologs are also present in other rhizobia and non-symbiotic bacteria, their function in *Sinorhizobium* appears to be specialized through both sequence variation and expression changes (DiCenzo et al. 2017), contributing to the adaption of the bacteria to terminal differentiation in galegoid legumes.

In addition to BacA, it has been shown that the high-molecular-weight exopolysaccharide succinoglycan produced by *S. meliloti* can also protect the bacteria from the antimicrobial action of the NCR247 peptide (Arnold et al. 2017, 2018). This finding aligns with previous transcriptional studies demonstrating that NCR247 induces the expression of *exo* genes, which encode proteins necessary for succinoglycan biosynthesis. Supporting this, some *exo* mutants exhibit increased sensitivity to the antimicrobial action of NCR247. These observations suggest that *S. meliloti* exopolysaccharides are critical not only during the early stages of nodule invasion but also during the late stage of symbiosis, where they protect the bacteria against the bactericidal action of cationic NCR peptides.

Rhizobia can modulate their developmental fate by actively degrading host-derived NCR peptides for their benefits. One such gene is the *host range restriction peptidase* (*hrrP*), located on a naturally occurring accessory plasmid (Price et al. 2015). This gene can disrupt terminal bacterial differentiation while enhancing bacterial proliferation within the nodule. The ability of *hrrP* to suppress nitrogen fixation is contingent on the genotypes of both the host plant and the rhizobial strain. The purified HrrP protein can degrade a range of NCR peptides encoded by *M. truncatula*.

Nitrogen fixation specificity has also been documented in non-galegoid legumes, such as soybeans and *Lotus* spp., where the plants do not encode NCR peptides and the bacterial partners do not undergo terminal differentiation (Fig. 2). In soybeans, it was reported that this type of incompatibility was associated with the induction of phytoalexin accumulation (Parniske et al. 1990; Pazdernik et al. 1997); however, molecular genetic studies are lacking in this aspect. In *Lotus japonicus*, a gene called *APN1*, which encodes a nodule-specific aspartic peptidase, has been identified as essential for nitrogen fixation in a strain-specific manner (Shimoda et al. 2020; Yamaya-Ito et al. 2018). The bacterial determinant of this specificity is an autotransporter, which is part of a protein secretion system in Gram-negative bacteria. The autotransporter secretes a part of its own protein (a passenger domain) into extracellular spaces, inducing the expression of genes involved in nodule senescence. The APN1 protein can cleave the passenger protein, thus suppressing the negative effects of the rhizobial autotransporter on symbiotic nitrogen fixation in root nodules.

## Overcoming host-range specificity is essential for engineering root nodule symbiosis in non-nodulating plants

Plant biologists have long sought to impart the ability for root nodule symbiosis to crops that do not naturally possess this trait. Recent advances in understanding the genetics and evolution of this process have raised optimism about achieving this goal (Huisman and Geurts 2019; Rogers and Oldroyd 2014; Yan and Bisseling 2024). The evolutionary origins of nodulation symbiosis remain debated: some researchers argue it evolved independently in different plant lineages, while others propose a single origin in a common ancestor of the Nitrogen-Fixing Clade, followed by multiple losses (Doyle 2011, 2016; Griesmann et al. 2018; Kates et al. 2024; Libourel et al. 2023; van Velzen et al. 2018). Nevertheless, the root nodule symbiosis shares a common symbiosis signaling pathway (CSSP) with the more ancient mycorrhizal symbiosis, which is present in both nodulating and non-nodulating plants. This suggests that the evolution of nodulation is built upon pre-existing plant developmental and symbiotic networks, along with the innovation of some key proteins, such as NIN (Nodule Inception), a transcription factor crucial for various steps in nodulation (Borisov et al. 2003; Fu et al. 2022; Marsh et al. 2007; Schauser et al. 1999) and RPG (Rhizobium Directed Polar Growth), a coiled-coil protein essential for the root hair infection process (Arrighi et al. 2008).

*NIN* expression, triggered by activation of the CSSP, is essential for both infection and nodule organogenesis. NIN is a founding member of a small family of NIN-like proteins (NLPs) that emerged from gene duplication before the evolution of the nitrogen-fixing clade (Liu and Bisseling 2020). In non-legumes, NLPs function as nitrate-sensing regulators (Konishi and Yanagisawa 2013; Liu et al. 2022b; Zhang et al. 2022). Some NLP family members are found in the cytoplasm under low or no nitrate conditions but move to the nucleus in response to higher nitrate levels (Durand et al. 2023; Lin et al. 2018; Marchive et al. 2013). In contrast, NIN is in the nucleus even when nitrate levels are low, indicating that its constant nuclear presence likely represents an evolutionary adaptation associated with its role in nodulation (Liu et al. 2021a).

Nodule organogenesis involves the recruitment of genes regulating lateral root development, such as LBD16, the PLETHORA transcription factors, and the auxin biosynthesis enzyme YUCCA (Du and Scheres 2017; Franssen et al. 2015; Schiessl et al. 2019; Soyano et al. 2019). Auxin can induce the formation of nodule-like structures in both legumes and non-legumes (Hirsch et al. 1989; Ridge et al. 1993). Recently, it has been demonstrated that nanobody-mediated heterodimer formation of the legume Nod factor receptors is sufficient to induce nodule organogenesis in the absence of rhizobia or Nod factors (Rübsam et al. 2023). Intriguingly, two barley LysM-RLK receptors, which are unable to perceive Nod factors, can also trigger nodule organogenesis when expressed in *Lotus* using a similar nanobody strategy (Rübsam et al. 2023).

In most legumes, rhizobial infection occurs through the formation and progression of intracellular infection threads (ITs), a process that requires RPG (Arrighi et al. 2008), VAPYRIN (VPY) (Murray et al. 2011), and a ubiquitin E3 ligase LIN (Kiss et al. 2009). RPG, VPY, and LIN form a protein complex essential for IT progression (Liu et al. 2019, 2021b; Lace et al. 2023; Li et al. 2023). Once inside the nodule cells, the bacteria are enveloped by a plant-derived membrane called the symbiosome membrane. This membrane is crucial for establishing an endosymbiotic relationship because it prevents direct exposure of the bacteria to the host cytoplasm, allowing the host to regulate nutrient transport to the bacteria. The formation of this symbiotic interface involves the interaction of specific v-SNARE proteins on transport vesicles with t-SNARE proteins on the target membrane, driving the fusion of the vesicle membrane with the target membrane (Lipka et al. 2007; Huisman et al. 2016).

Engineering the above-mentioned pathways through gene transfer or gene editing could lead to the development of nodules or nodule-like structures on non-legume roots. A significant challenge is to make these nodules functional by ensuring they can be effectively infected and colonized intracellularly by nitrogen-fixing rhizobia. Besides, the pathways needed for infection and accommodation of compatible rhizobia, researchers must also make the non-host plants susceptible to rhizobia infection. This process is complicated by the highly specialized interactions between rhizobia and their natural host plants, which have evolved intricate mechanisms for recognizing and establishing symbiosis. Beyond Nod factor recognition, rhizobia must evade or suppress non-host defense responses triggered by various effectors and molecular patterns. Thus, achieving functional root nodule symbiosis in non-legumes requires a multifaceted approach that integrates advances in plant genetics, microbial physiology, and symbiotic signaling.

## Conclusion and future perspectives

Host range specificity is a hallmark trait of root nodule symbiosis. Genetic and molecular mechanisms regulating the recognition specificity are diverse, involving multiple host and bacterial signals. These specialized interactions result from the long-term coadaptation of hosts and their microsymbionts, shaped by their ecological niches. Knowledge of the genetic foundations of symbiotic specificity is crucial for selecting or engineering the host and/or bacteria to overcome host range limitations and enhance nodulation and nitrogen fixation efficiency. This knowledge will also help achieve a long-held goal of plant biologists: transferring the nitrogen-fixing symbiosis to non-leguminous crops as overcoming host range barriers beyond Nod factor perception is necessary. To reach this goal, research is needed to elucidate the genetic pathways in the host, the molecular signals in the bacteria, and to characterize how plants perceive and transmit bacterial signals, ultimately leading to strain-specific nodulation.

## Data Availability

Data sharing not applicable to this article as no datasets were generated or analysed during the current study.
